# Insights into the interaction between time and reward prediction on the activity of striatal tonically active neurons: A pilot study in rhesus monkeys

**DOI:** 10.14814/phy2.70037

**Published:** 2024-09-08

**Authors:** A. C. Martel, P. Apicella

**Affiliations:** ^1^ Institut de Neurosciences de la Timone, UMR 7289 Aix Marseille Université, CNRS Marseille France; ^2^ Present address: Emory National Primate Research Center Emory University Atlanta Georgia USA

**Keywords:** acetylcholine, basal ganglia, interval timing, motivation

## Abstract

Prior studies have documented the role of the striatum and its dopaminergic input in time processing, but the contribution of local striatal cholinergic innervation has not been specifically investigated. To address this issue, we recorded the activity of tonically active neurons (TANs), thought to be cholinergic interneurons in the striatum, in two male macaques performing self‐initiated movements after specified intervals in the seconds range have elapsed. The behavioral data showed that movement timing was adjusted according to the temporal requirements. About one‐third of all recorded TANs displayed brief depressions in firing in response to the cue that indicates the interval duration, and the strength of these modulations was, in some instances, related to the timing of movement. The rewarding outcome of actions also impacted TAN activity, as reflected by stronger responses to the cue paralleled by weaker responses to reward when monkeys performed correctly timed movements over consecutive trials. It therefore appears that TAN responses may act as a start signal for keeping track of time and reward prediction could be incorporated in this signaling function. We conclude that the role of the striatal cholinergic TAN system in time processing is embedded in predicting rewarding outcomes during timing behavior.

## 1 INTRODUCTION

Convergent evidence indicates that the striatum, the main recipient of afferents to the basal ganglia, is a key component in brain networks involved in temporal processing. A deficit in timing behavior has been observed in pathological conditions that affect the striatum and its dopaminergic input from the midbrain, as in Parkinson's disease (Parker et al., 2013; Pastor et al., 1992) and attention‐deficit hyperactivity disorder (Noreika et al., 2013; Rubia et al., 2009). The role of the striatum and the modulating influence of dopamine in timing processes is also supported by brain imaging studies in humans (Coull et al., 2011; Harrington et al., 2004) and lesion/inactivation studies in animals (Meck 1996, 2006; Mello et al., 2015; Paton & Buonomano, 2018; Soares et al., 2016). In addition, previous electrophysiological studies in animals performing timing tasks have reported changes in striatal neuronal activity that could convey information about duration in the range of seconds, at both single‐neuron and population levels (Bakhurin et al., 2016, 2017; Chiba et al., 2015; Gouvêa et al., 2015; Jin et al., 2009; Matell et al., 2003; Wang et al., 2018; Zhou et al., 2020).

Up to now, hypotheses about the neuronal basis of striatal timing function have focused on the main population of neurons in the striatum, the GABAergic projection neurons (Coull et al., 2011; Mello et al., 2015; Merchant et al., 2013). Striatal output pathways are regulated by local circuit neurons, notably cholinergic interneurons which interact closely with dopaminergic terminals in the striatum (Cachope et al., 2012; Cai & Ford, 2018; Threlfell et al., 2012; Threlfell & Cragg, 2011). A link has been proposed between cholinergic transmission and timing behavior based on lesion and pharmacological studies in rodents (Buhusi & Meck, 2005; Meck, 1996), but none of these studies were specific enough to determine the exact contribution of the intrastriatal cholinergic innervation to the processing of time. Recently, a study in rats using an immunotoxin approach for selective inactivation of cholinergic interneurons from the dorsal striatum has provided evidence that they are critical for the memory representation of durations (Nishioka & Hata, 2024).

In behaving rodents and primates, single‐neuron recordings of tonically active, putative cholinergic interneurons (TANs) have been conducted primarily in relation to motivation and reinforcement learning (Apicella, 2007). It has been reported that most of the TANs show brief depressions of their tonic firing in response to motivationally salient stimuli and this responsiveness appears to be strongly influenced by expected event timing (Ravel et al., 2001; Sardo et al., 2000). These time‐dependent changes in TAN activity have led us to emphasize the notion of a relationship of the TAN system to temporal aspects of stimulus prediction (Apicella et al., 2006). However, this role was studied during the performance of simple conditioning tasks, such as reaction time tasks and Pavlovian protocols, and no study to date has specifically investigated TAN activity in animals performing a task that has precise timing requirements. In this regard, the possibility of an integration of temporal information in the modulation of TAN activity is still an open question. In addition, it is increasingly recognized that time estimation and expectation about reward are concurrently active during tasks used to elicit timing behavior (Fung et al., 2021; Kirkpatrick, 2014), and the TAN network might potentially be involved in these two closely intertwined processes.

The present study aimed to examine the activity of TANs in rhesus monkeys performing a reaching task that has precise timing requirements, with the expectation of receiving a reward for completing correctly timed movements. We found that TANs were modulated in response to the stimulus that contains specific information for allowing monkeys to adjust the timing of their movements and these modulations may also integrate the estimated probability of receiving a reward upon completion of correctly timed movements. These findings provide evidence for the view that local cholinergic circuitry can be considered as an important element of striatal processing involved in controlling timed behaviors within a rewarding context. A preliminary account of this work previously appeared in a short review (Martel & Apicella, 2021).

## 2 MATERIALS AND METHODS

### 2.1 Animals and recording procedures

Experiments were conducted on two adult male rhesus monkeys (*Macaca mulatta*), C and D, weighing 11 and 7 kg, respectively, kept in pairs in their home cage. This study was performed in accordance with the principles of the European Union Directive 2010/63/EU on the protection of animals used for scientific purposes and all procedures were approved by the Ethics Committee of the Institut de Neurosciences de la Timone (protocol #3057–2,015,120,809,435,586). Prior to the experiments, each monkey was implanted with a head‐holding device and recording chamber (25 × 35 mm) under general gas anesthesia (sevoflurane 2.5%) and aseptic conditions. The center of the chamber was stereotaxically directed to the anterior commissure (AC) based on the atlas of Paxinos et al. (Paxinos et al., 2008). The animals received antibiotics and analgesics for a period of 5 days after the surgery.

Recordings were obtained with glass‐coated tungsten electrodes (impedance: 2–3 MΩ) passed inside a guide tube (0.6 mm outer diameter) and lowered to the striatum with a manual hydraulic microdrive (MO‐95; Narishige, Tokyo, Japan). Electrode tracks were made vertically into regions of the striatum anterior and posterior to the AC, mostly in the putamen.

Neuronal activity was amplified (×5000), bandpass‐filtered (0.3–1.5 kHz), and spike sorting was performed on‐line using a window discriminator (NeuroLog; Digitimer, Hertfordshire, UK). Continuous monitoring of the spike waveform on a digital oscilloscope allowed us to check the isolation quality of the recorded neurons. Recordings took place in the left striatum and all reach movements were made with the arm contralateral to the recorded hemisphere. In line with many previous single‐neuron recording studies in the striatum of behaving monkeys, TANs were electrophysiologically identified by waveform shape, firing rate, and response to task stimuli consisting of a transient decrease in activity. A computer controlled the behavioral task and data acquisition using a custom‐made software developed by E. Legallet under LabVIEW (National Instruments).

### 2.2 Behavioral task

**FIGURE 1:**
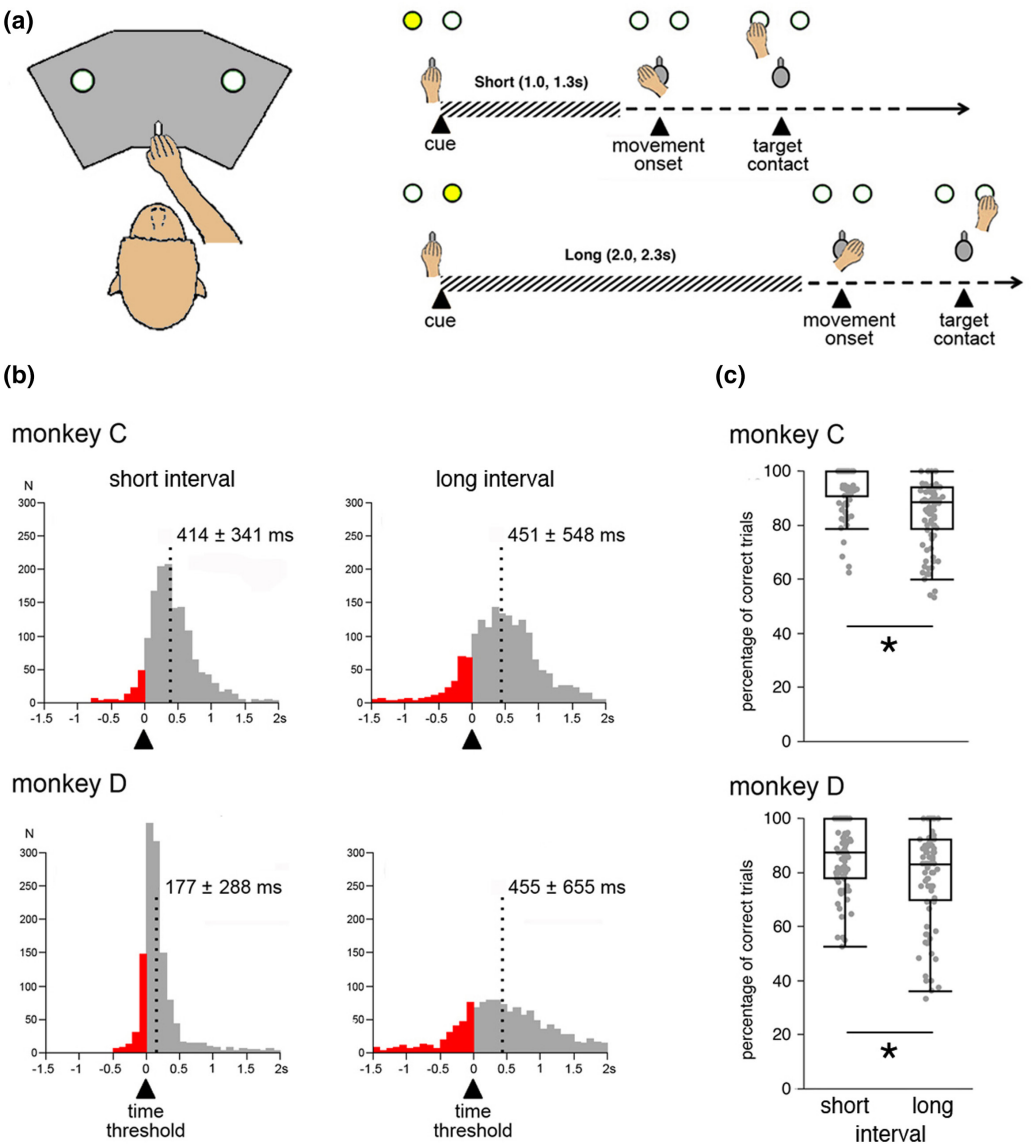
Self‐timed reaching task and timing performance. (a) Temporal sequence of events in the task. Monkeys were required to initiate reaching movement after a specified time interval has elapsed. At the beginning of the trial, a visual cue (yellow light), either on the left or the right side, indicated the duration of the interval (*Short* or *Long*) in the range of seconds, each time interval being associated with a particular spatial location of the stimulus. Hatched horizontal lines indicate the minimum waiting period before movement initiation assigned to each location of the cue (time threshold). Correctly timed movements were reinforced with fruit juice immediately after target contact. (b) Distribution of movement onset times produced by monkeys in the task. Red parts of the histograms represent trials where movements started before reaching the time threshold (underestimation errors). The spread of the response distribution is proportional to the length of the interval to‐be‐timed, consistent with the scalar property of interval timing. Values correspond to the mean of the distributions ± SD. Dashed lines indicate the mean. Number of trials were 1370 and 1594 for monkey C and 1219 and 1146 for monkey D, for short‐ and long‐interval trials, respectively. (c) Reward frequency during task performance. Box plots of the percentage of correctly timed movements. Each point corresponds to the proportion of correct movements from the short‐ and long‐interval trials of a block. Boxes indicate 25–75 percentile ranges of the distributions and lines through boxes correspond to medians. Isolated dots indicate outliers. Percentage of correct movements differed between time intervals for both monkeys (*p < 0.01, paired *t*‐test).

Training and recording sessions took place in a setup similar to that described previously (Marche & Apicella 2017). Monkeys were seated in a restraining box facing a vertical panel containing two metal knobs (diameter, 10 mm) serving as movement targets, positioned 18 cm apart at the animal's eye level, and two light‐emitting diodes (LEDs), one above each target. A resting bar was mounted in the lower part of the panel and a tube was positioned directly in front of the monkey's mouth for dispensing drops of fruit juice (0.3 mL) as a reward. Animals were trained in a time estimation task (TET) in which one of the two LEDs was lit as a cue indicating both the target of movement and the minimum waiting period before initiating a reaching movement. A schematic representation of the sequence of events in the TET is given in Figure 1a. The trial began when the monkey kept its hand on the bar. After 1 s, a LED was illuminated either on the left or on the right side, indicating a *short* or *long* waiting period from the onset of the visual stimulus, respectively. The cue location was pseudorandomly selected from trial to trial. In all cases, the cue was presented for 0.5 s and was followed by a delay (short or long), the length of the waiting period being defined from the cue onset. Although we employed the same training procedure for both monkeys, we experienced difficulty in training monkey D to estimate duration longer than 2 s which forced us to use shorter durations (1.0 and 2.0 s), compared to monkey C (1.3 and 2.3 s). Correct performance of the task required that monkeys released the bar after the time threshold was reached (limited to 2 s after the criterion time) and made adequate target contact. Each trial lasted 6 s, so that the overall temporal structure of the task remained the same regardless of the interval duration. If the monkey released the bar before reaching the time threshold or touched the target in more than 1 s after bar release, it was not rewarded. In these cases, the trial continued until the end of its total duration, and the same trial was repeated until a rewarded movement was successfully completed. Trials on which animals did not react after cue onset or did not touch a target after bar release were excluded from the analyses. Both monkeys received training on the TET until they reached a stable level of performance corresponding to a criterion of at least 75% correctly performed trials. Because the location of the cue indicated both the interval duration and the spatial location of the movement target, we attempted to reverse the relationship between cue location and interval duration (i.e., left/long and right/short instead of left/short and right/long). Unfortunately, when we introduced this change in the experimental design, it took a lot of trials over several test sessions before animals reinstated stable performance, possibly because of previous extensive training with the same cue‐interval condition which may have compromised flexible remapping. Thus, we did not use this task variant which was not compatible with our electrophysiological analysis.

We also tested in a Pavlovian conditioning task (PCT) the effects of intervals in a time range similar to that used in the TET, so that to exclude possible confounding factors that were linked to target reaching. In the PCT, access to the working panel was prevented by closing the sliding door at the front of the restraining box and monkeys remained motionless with their arms relaxed in a natural position. Animals were then exposed to a visual stimulus (red light, 0.5 s duration), presented either on the left or right side, whose onset was initiated by the experimenter. This cue was followed by the delivery of reward after a fixed interval of 1.0 or 2.0 s, depending upon stimulus location. The PCT matched the TET in terms of location‐interval combinations (left/short and right/long) and total trial duration (6 s). During PCT sessions, the licking movements of the monkeys were monitored using force transducers (strain gauges) attached to the tube delivering liquid. Signals from the strain gauge device were digitized at 500 Hz and stored into an analog file.

### 2.3 Data analysis

Performance in the TET was assessed by measuring the time between the onset of the cue and bar release (movement onset time, MOT), for each time‐interval combination. We examined MOT distributions to determine whether the spread of these distributions is proportional to the length of the interval being estimated. Neurons were determined to be task‐responsive according to methods similar to those described previously (Marche et al., 2017). Briefly, a “sliding window” analysis was used to quantify the time course of changes in TAN activity after a specific task event. A test window of 100 ms duration was advanced in 10 ms increments across the trial, starting at cue onset, movement onset, or target contact. The average spike counts in the test window was compared, at each step, with that calculated during the 500 ms immediately preceding the onset of the cue (control period). The onset of a modulation was taken to be the beginning of the first of at least five consecutive steps showing a significant difference (Dunn's test for multiple nonparametric comparisons, *p* < 0.01) as against the activity in the control period.

Quantitative analysis of spike rates was also performed for windows with fixed durations that were specifically determined for each monkey on the basis of the analysis of latency and duration of statistically significant decreases in activity (i.e., pauses) detected with the sliding window procedure. We set these time windows such that they included most of the pause onset and offset times. According to this analysis, time windows after the cue onset were 93–309 ms for both short‐ and long‐interval trials, for monkey C, and 127–313 ms and 211–418 ms for short‐ and long‐interval trials respectively, for monkey D. The windows determined by the average onset and offset times of pause responses to reward were 139–369 ms and 194–391 ms for both short‐ and long‐interval trials, for monkey C and D, respectively. We then rated the magnitude of activity changes in each time window for each neuron and each time interval, irrespective of whether neurons were individually responsive or not. We set quantification windows in the same way for assessing the magnitude of TAN activity after the stimulus and reward in the PCT. The percentages of TANs showing pause responses in relation to the total number of neurons tested were calculated for each condition and differences in proportions of responding neurons among conditions were statistically assessed with the *χ*2 test. Differences in the magnitude of changes in activity were assessed with a Wilcoxon rank‐sum test.

### 2.4 Recording sites

The location of individual recorded neurons was confirmed histologically in both monkeys. Near the end of the experiments, small electrolytic lesions (20 μA for 30 s, cathodal current) were made at several points along selected electrode tracks. The monkey was deeply anesthetized with pentobarbital and perfused through the heart with 0.9% saline followed by 4% paraformaldehyde (pH 7.4 phosphate buffer). Frozen sections (40‐μm thick) were made in the frontal plane and stained with cresyl violet. We then reconstructed the location of each recorded neuron according to the depth and coordinates of electrode penetrations based on the retrieved sites of marking lesions. In line with previous work, we used the AC as a structural boundary separating the striatum into motor and associative parts. Based on the reconstruction of recording sites, TANs were sampled between 5 mm anterior and 4 mm posterior to the AC, mainly over the medio‐lateral extent of the putamen.

## 3 RESULTS

### 3.1 Timing behavior

Both monkeys performed self‐initiated movements whose onset time variability increased with the duration of the interval to be estimated, as reflected by a broader distribution of the MOTs around the time threshold for long‐interval trials compared to short‐interval trials (Figure 1b). This indicates that timing accuracy was lower as interval duration was extended, consistent with the scalar property of interval timing described in both animals and humans in a variety of tasks (Gibbon, 1977). It is noteworthy that the distribution of MOTs produced by monkey D in short‐interval trials was strongly skewed toward the time threshold for initiating movement, possibly reflecting a particular response initiation strategy applied in these trials. We quantified monkeys' accuracy of timed movements by calculating the proportions of rewarded trials (i.e., movements made after reaching the time threshold). Figure 1c represents the distribution of monkeys' correctly timed movements for each interval duration across trial blocks. The reward rate achieved over the blocks was higher in monkey C (89 ± 11%) than in monkey D (82 ± 17%) (Student *t*‐test, *t* = 4.852, *p* < 0.0001) and both monkeys were more successful in obtaining rewards in short‐interval trials than in long ones (monkey C: *t* = 5.418, *p* < 0.0001; monkey D: *t* = 3.096, *p* < 0.002). Overall, behavioral data revealed that both monkeys could adjust the timing of their movements according to temporal information contained in the cue, with a lower accuracy of estimated elapsed time for the long interval, compared to the short one.

### 3.2 Identification of TANs

**FIGURE 2:**
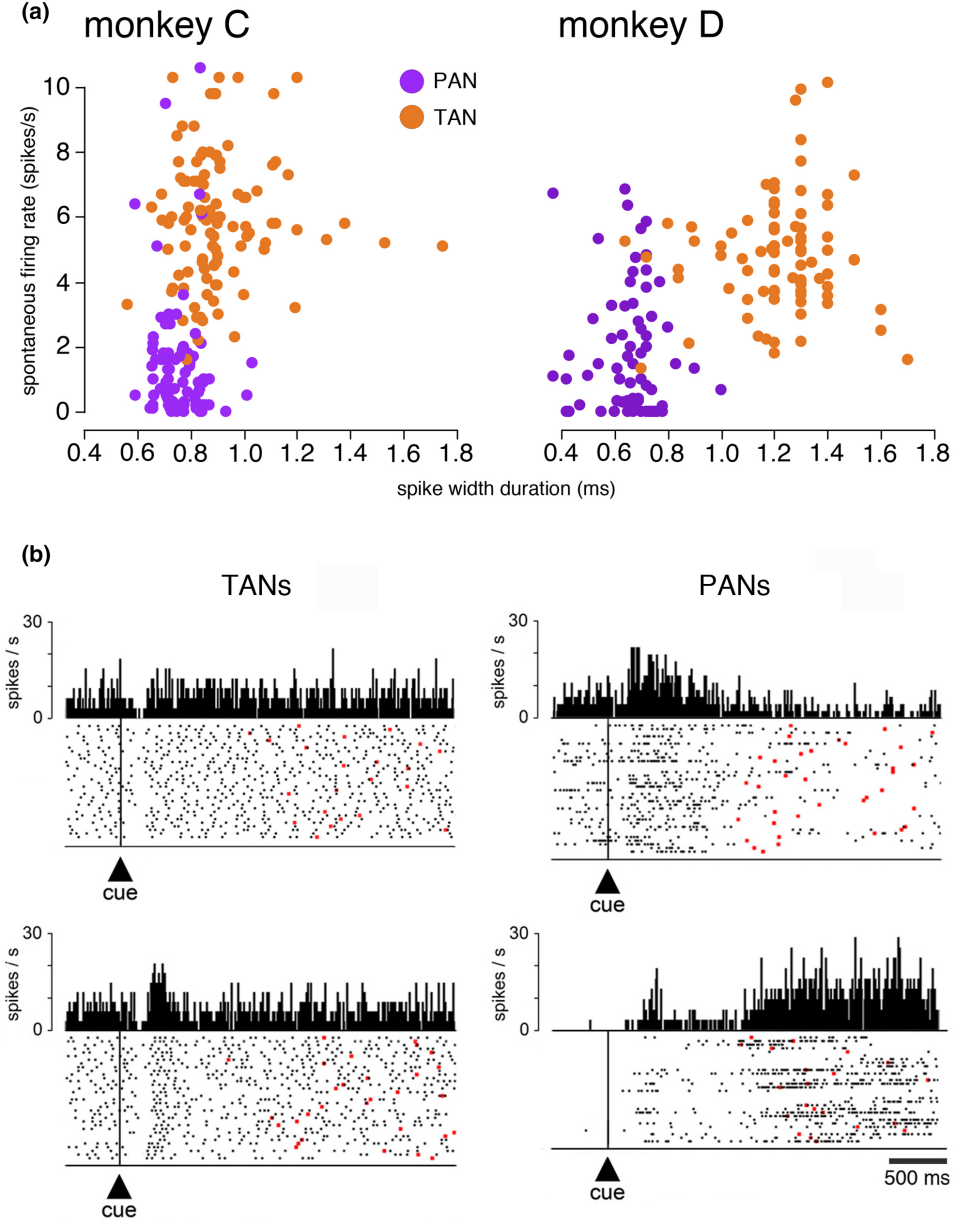
Electrophysiological identification of TANs. (a) Scatterplot of the baseline firing rate and spike width duration for the two main striatal neuron classes. Each dot represents data from an individual neuron. TANs displayed higher spontaneous discharge rate and longer spike duration than phasically active neurons (PANs). (b) Two examples of task‐related activity modulation for each type of neuron. Each trial is displayed as a row of spikes (dots) with perievent time histogram above each raster plot. Activity is referenced to cue onset which is marked by vertical lines. The sequence of trials is shown chronologically from top to bottom in each raster plot, irrespective of the duration of the interval. Movement onsets are indicated by red dots.

We recorded the activity of 200 neurons (114 in monkey C, 86 in monkey D) identified as tonically active neurons (TANs), presumed cholinergic interneurons, on the basis of their spiking features and characteristic changes in activity in response to motivationally salient stimuli (Apicella, 2017). As shown in Figure 2a, the TANs had a mean baseline activity which was higher than that of striatal projection neurons, referred to as physically active neurons (PANs) (Monkey C: TANs: *n* = 108, 5.94 ± 1.94 spikes/s; PANs: *n* = 86, 1.47 ± 1.91 spikes/s; Monkey D: TANs: *n* = 86, 4.82 ± 1.72 spikes/s; PANs: *n* = 68, 1.83 ± 1.92 spikes/s). Their mean spike width duration was larger than that of the PANs (Monkey C: TANs: *n* = 108, 905 ± 169 s; PANs: *n* = 86, 758 ± 80s; Monkey D: TANs: *n* = 85, 1220 ± 194 s; PANs: *n* = 68, 656 ± 114 ms). As described in prior studies (Marche & Apicella, 2017), task‐related changes in firing served as another criterion to identify TANs and distinguish them from PANs. Figure 2b shows two examples of neurons categorized as TAN or PAN. As is apparent in the left part of the figure, the predominant task‐related TAN modulation consisted in a transient depression in firing (or *pause*) in response to the cue (76 of 200 neurons, 38%). In the example TAN at the bottom, the pause response was followed by a transient increase in firing, designated as the *rebound*, which occurred less frequently (23 of 200 neurons, 12%). A brief activation preceding the pause occurred in only three neurons. In the present study, we focused on the stereotyped pause in TAN firing which was the most frequently observed response component. Unlike characteristic depressions in firing seen in TANs, PANs exhibited heterogeneous profiles consisting in transient or sustained increases in activity occurring at particular periods of task performance. On the two examples shown in the right part of Figure 2b, one PAN was preferentially activated after the onset of the cue, whereas the other displayed an increase in activity persisted in some trials until the movement was initiated. In practice, as previous studies have shown, spiking features and functional characterization of TANs allowed their clear differentiation from PANs in the striatum.

### 3.3 Sensitivity of TANs to the cue

**FIGURE 3:**
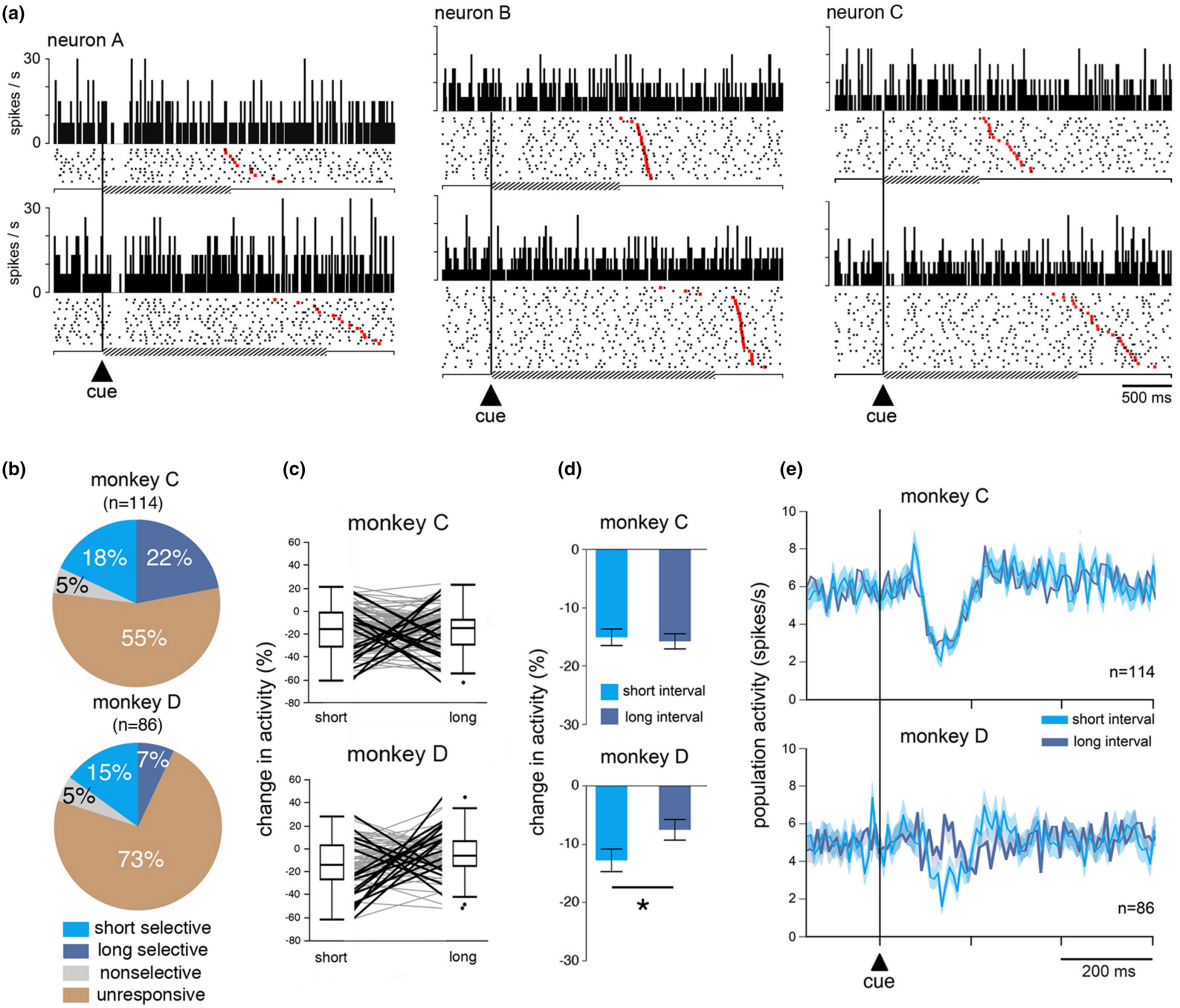
Sensitivity of TANs to the timing cue. (a) Three example neurons responding to the cue. Left, nonselective response; Middle, short selective response; Right, long selective response. Same conventions as in Figure 2b, except that data are separated by interval duration and sorted by movement onset time. Hatched horizontal lines indicate the minimum waiting period before initiating the movement (time threshold). A few underestimation errors are visible at the top of each raster (red markers before reaching the time threshold). (b) Relative proportions of neurons responding to the cue in short‐ and/or long‐interval trials. The percentages were calculated from the total number of recorded neurons (*n*) in each monkey. (c) Changes in TAN activity after cue onset separately for short‐ and long‐interval trials for each neuron recorded. Each line indicates the data of one neuron. Thick lines indicate significant differences in the magnitude of changes in activity between time intervals (*p* < 0.05, Wilcoxon signed‐rank test). Boxes indicate 25–75 percentile ranges of the distributions and lines through boxes correspond to medians. Isolated dots indicate outliers. (d) Comparison of magnitudes of changes in TAN activity after the cue separately for the short and long time intervals. Data are indicated as decreases in percentage below baseline activity and expressed as means ± SEM, *p < 0.05 (Wilcoxon signed‐rank test). (e) Modulation of TAN responses to the cue, shown as population averages separately for the short and long time intervals. The averaged population activity is aligned on the cue onset marked by the vertical line and included both correctly timed movements and underestimation errors. Colored curves represent mean activity of TANs separately for short‐ and long‐interval trials calculated in nonoverlapping time bins of 10 ms. Shading indicates SEM. n, number of neurons included for population curves.

For each recorded TAN, we analyzed the responsiveness to the cue separately for short and long intervals. As mentioned before, statistically significant changes in TAN activity relative to the baseline mainly consisted of a pause response to the onset of the cue associated with the short and/or long interval (monkey C: 51 of 114 neurons, 45%; monkey D: 23 of 86 neurons, 27%), the difference in the fraction of responsive neurons being significant between monkeys (*χ*2 = 6.80, *df* = 1, *p* = 0.009). Example responsive neurons are shown in Figure 3a. The first neuron (left) displayed a response to the cue for the short‐ and long‐interval trials, while in the other two neurons, the response to the cue was most prominent for only one interval, either short (middle) or long (right). To explore the role of TANs in temporal information processing, we examined selectivity of changes in their activity for one time interval over the other. Among all responsive neurons (*n* = 74), 10 (14%) responded to the cue regardless of the interval and 64 (86%) responded to the cue for only one interval, indicating that most TAN responses were specific for one interval duration (Figure 3b). There were no significant differences in the fraction of TANs showing selectivity for one interval or the other in monkeys C (*χ*2 = 0.69, *df* = 1, *p* = 0.405) and D (*χ*2 = 2.89, *df* = 1, *p* = 0.08). To further analyze whether the sensitivity of TANs to the cue differed according to interval duration, we assessed the level of activity for each recorded neuron during specific time windows which we selected on the basis of our analysis of latency and duration of pause responses for each monkey and each time interval (see Materials and Methods). We then rated the magnitude of change after the cue onset in every neuron, regardless of a significant modulation of activity relative to the baseline, and we examined, for each neuron, whether the activity differed between the short‐ and long‐interval trials (Wilcoxon rank‐sum test, *p* < 0.05). As illustrated in Figure 3c, we found that the number of TANs showing stronger depression in firing for one time interval or the other was roughly the same in monkey C. Out of the 114 neurons recorded in this animal, 17 (14.9%) showed activity that differed significantly between the two intervals, eight neurons showing greater modulation on short trials than on long trials, and nine neurons showing the opposite. On the other hand, a higher number of TANs were modulated more strongly for the short interval than for the long one in monkey D. Among the 74 neurons recorded in this animal, significant differences in activity was found in 16 neurons (21.6%), neurons showing a stronger modulation of their activity being twice as frequent on short‐interval trials (*n* = 11) as on long‐interval trials (*n* = 5). These patterns of modulation correspond well to the results obtained when all recorded neurons were pooled (Figure 3d), the responsiveness of TANs to the cue remaining the same in terms of magnitude for short and long intervals in monkey C (Wilcoxon rank‐sum test, *z* = 0.415, *p* = 0.677), whereas it was greater for the short interval than for the long one in monkey D (Wilcoxon rank‐sum test, *z* = 2.241, *p* = 0.025). This was also reflected in the average activity from the whole population of TANs recorded in each animal (Figure 3e) revealing a strong depression in firing following the presentation of the cue for both intervals in monkey C, whereas it was only apparent for the short interval in monkey D.

To summarize, monkey C showed a consistent level of TAN responsiveness to the cue which was not modified in conjunction with interval duration, whereas TANs demonstrated higher sensitivity for the cue associated with the short interval in monkey D, indicating that TAN responses to the cue may discriminate between the two time intervals. In this latter animal, the behavioral findings showed that the onset of movement in short‐interval trials was remarkably close to the time threshold, providing support to the idea that differences in the responsiveness of TANs to the cue may reflect adjustments of motor reactions based on the time elapsed from cue onset.

### 3.4 Sensitivity of TANs to reward

In the task we used, the cue not only contains temporal information, but also predicts reward for correctly timed movements. Given that TAN activity was reported to be sensitive to predictions about the probability and timing of rewards, it is possible that TAN responses to the cue were influenced by the overall reward rate achieved in the TET. To test whether reward prediction could be integrated in TAN responses, we examined the responsiveness of TANs to the reward itself. It was reasoned that a higher reward‐predictive value of the cue would decrease the responsiveness to reward, consistent with the inverse relationship between reward prediction and TAN responses to reward we have reported in previous studies (Apicella et al., 2009; Ravel et al., 2001).

**FIGURE 4:**
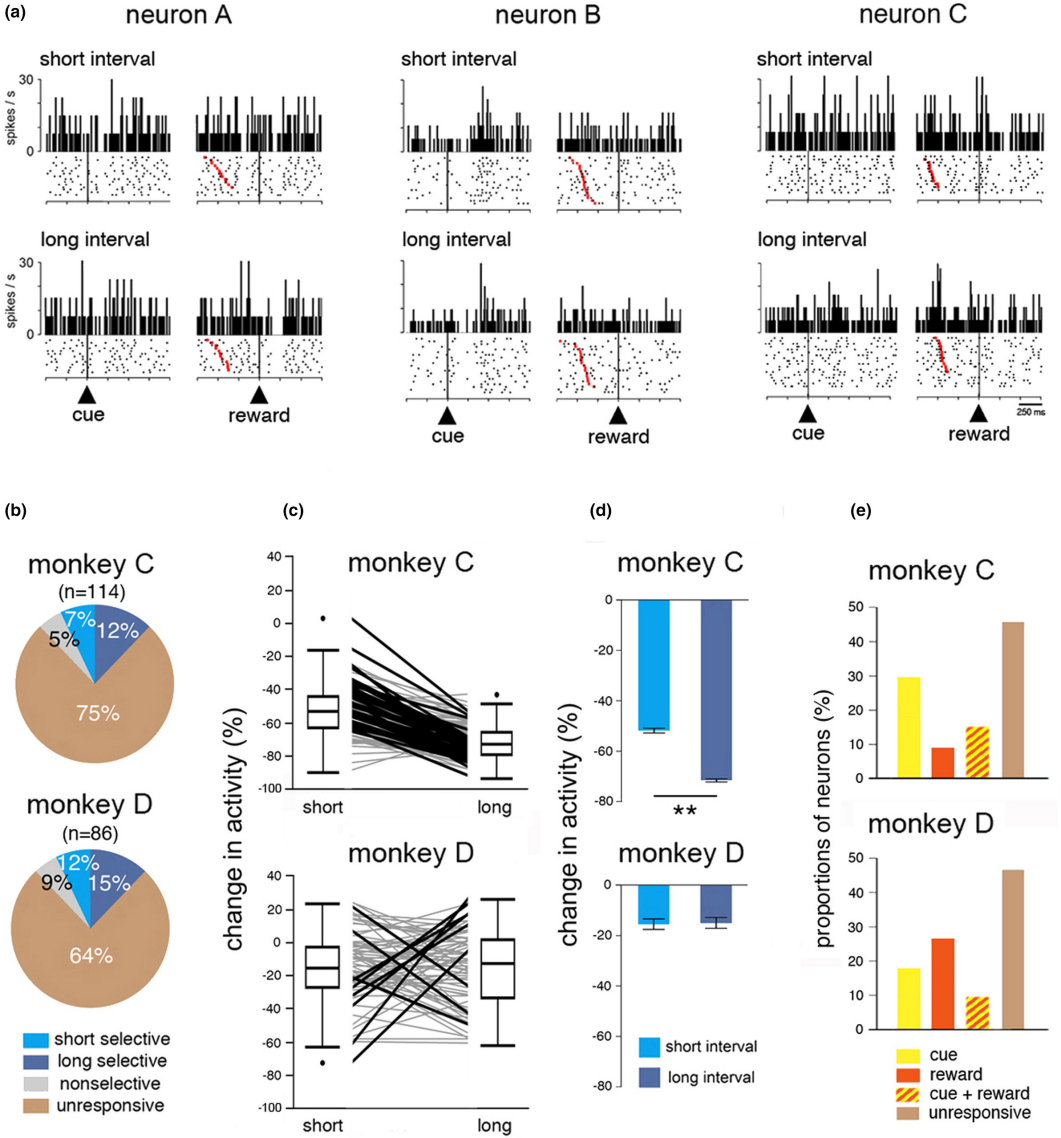
Sensitivity of TANs to reward. (a) Three example neurons responding to the cue and/or reward. Same conventions as in Figure 3a, except that activity is separately aligned on cue onset and reward delivery. A few underestimation errors are visible at the bottom of each raster aligned on reward (absence of red marker). (b) Percentages of different response types evoked by reward delivery. Same conventions as in Figure 3b. (c) Changes in TAN activity after cue onset separately for short‐ and long‐interval trials for each neuron recorded. Same conventions as in Figure 3c. (d) Comparison of magnitudes of changes in TAN activity after the delivery of reward separately for the short and long time intervals. Same conventions as in Figure 3c, except that only rewarded trials were included. **p < 0.01 (Wilcoxon signed‐rank test). (e) Relative proportions of TANs responding or not to the cue and/or reward.

Of the 200 TANs studied, 79 responded to the delivery of reward in short‐ and/or long‐interval trials (monkey C: 28 of 114 neurons, 25%; monkey D: 31 of 86 neurons, 36%). Figure 4a shows examples of TANs responding to the cue with or without additional responses to reward. Some neurons responded both to the cue and reward (*left*), whereas other neurons displayed responses specifically related to the cue (*middle*) or reward (*right*). Assuming that reward prediction varies as a function of the difficulty of timing (i.e., the more difficult the interval to be timed, the lower the probability of reward), we first examined whether the selectivity of TAN responses to reward was higher for the long interval than for the short one. As shown in Figure 4b, differences in the fraction of responses to reward for the short and long intervals were not significant in both monkeys (monkey C: *χ*2 = 1.811, *df* = 1, *p* = 0.178; monkey D: *χ*2 = 0.452, *df* = 1, *p* = 0.501), suggesting that TANs did not display differential responsiveness to reward according to interval duration. We next assessed the impact of interval duration on TAN activity following reward within time windows adjusted to latency and duration of individual TAN responses to reward, for each interval and each monkey. We undertook a similar analysis to that done for the cue. Namely, for each neuron, we rated the magnitude of changes following reward in individual TANs according to the interval duration. As is apparent in Figure 4c, we found a clear difference in the magnitude of the TAN modulation following reward for 41 of 114 neurons (35.9%) in monkey C, all of them showing greater modulation for reward delivered in long‐interval trials. On the other hand, the responsiveness of TANs to reward differed between the two intervals in only 9 of 74 neurons (12.1%) in monkey D, with similar frequencies of neurons preferring the short (*n* = 6) or long interval (*n* = 3). This is consistent with the results of the analysis for the whole population of TANs, shown in Figure 4d, which revealed that the magnitude of reward response was stronger for the long interval than for the short one in monkey C (Wilcoxon rank‐sum test, *z* = 16.30, *p* < 0.0001), but did not differ significantly between intervals in monkey D (Wilcoxon rank‐sum test, *z* = 0.21, *p* = 0.833). Finally, as we pointed out in the example neurons for which activity is shown in Figure 4a (middle and right), TANs may respond selectively to the cue or to reward. Given that increasing sensitivity of TAN responses to the cue may be potentially associated with decreasing sensitivity of TAN responses to reward, as a reflection of reward prediction, we examined the proportions of selective responses (Figure 4e) and found that cue selective responses were more numerous than reward selective responses in monkey C (*χ*2 = 14.64, *df* = 1, *p* = 0.0001), whereas they did not differ significantly in monkey D (*χ*2 = 2.16, *df* = 1, *p* = 0.141). This result argues in favor of an influence of reward prediction in monkey C, but not in monkey D.

### 3.5 Influence of movement timing on TAN responsiveness

**FIGURE 5:**
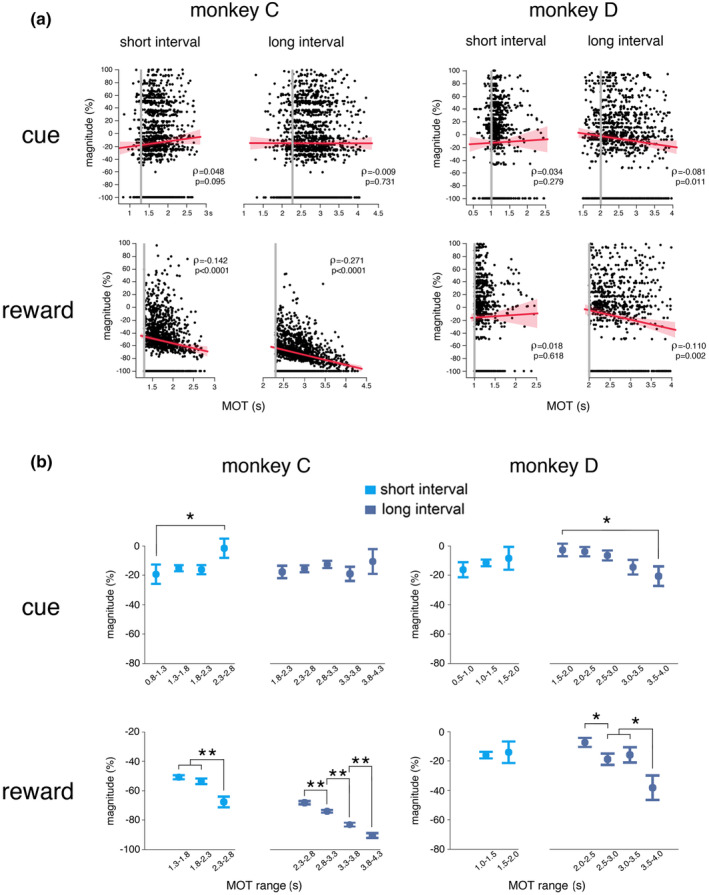
Influence of movement timing on TAN responsiveness. (a) Scatter plots of the magnitude of TAN modulations after the cue and reward versus the movement onset time (MOT), separately for the short and long intervals. The red lines indicate the fit of a linear regression and shading indicates the 95% confidence interval from the regression. Spearman correlation coefficient. Vertical gray lines indicate time thresholds (monkey C: 1.3–2.3 s; monkey D: 1–2 s). (b) Magnitudes of TAN responses to the cue computed separately from trials with MOTs within consecutive 500‐ms periods. Plots show, separately for the two interval durations, mean magnitude of TAN responses to the cue and reward, according to the range of MOT values. Error bars represent SEMs. *p < 0.05, **p < 0.01 (Wilcoxon signed‐rank test).

To test whether the modulation of TAN activity after the cue and reward could be related to the time at which the movement was initiated, we conducted a linear regression analysis of the level of TAN modulation in relation to the MOT produced by monkeys in the task. We performed this analysis on magnitudes calculated in the previously determined standard time windows. As shown in Figure 5a, we did not find any significant correlation between the magnitude of TAN modulations after the cue onset and MOTs, except for monkey D in which the strength of the depression in TAN firing was greater when the MOT was longer and this effect occurred only for the long interval. On the other hand, the magnitude of modulations following reward delivery showed a consistent relation to the MOT for both intervals in monkey C, being stronger when the MOT was longer. The same effect was observed in monkey D only for the long interval, but the correlation was not as strong compared with that observed in monkey C.

The relationship between the modulation of TAN activity and the timing of movement was further characterized by computing the magnitude of the depression in firing in successive MOT ranges. As illustrated in Figure 5b, a significant modulation of the TAN activity in relation to the timing of movement was found in monkey C, in which the responsiveness of TANs was stronger for early movement initiation for the short interval, and in monkey D, in which the responsiveness of TANs was stronger for late movement initiation for the long interval. This can be taken as evidence that the TAN response to the cue was influenced by some aspects of temporal processing, namely the time spent before making the movement. In addition, we observed a consistent and homogeneous enhancement of the TAN response to reward with later movement onsets, except in monkey D for the short interval.

In summary, we showed that the response of TANs to the cue could be influenced in distinct ways by the precise time at which the movement was initiated, depending on the monkey and interval duration. This can be interpreted as evidence for the notion that the TAN response to the cue may act as a signal initiating the timing process. On the other hand, we found that the reward elicited a more homogenous and larger TAN response when the timing of movements was delayed after reaching the time threshold, likely reflecting a decreasing reward prediction when movements were initiated late.

### 3.6 Influence of timing accuracy on TAN responsiveness

**FIGURE 6:**
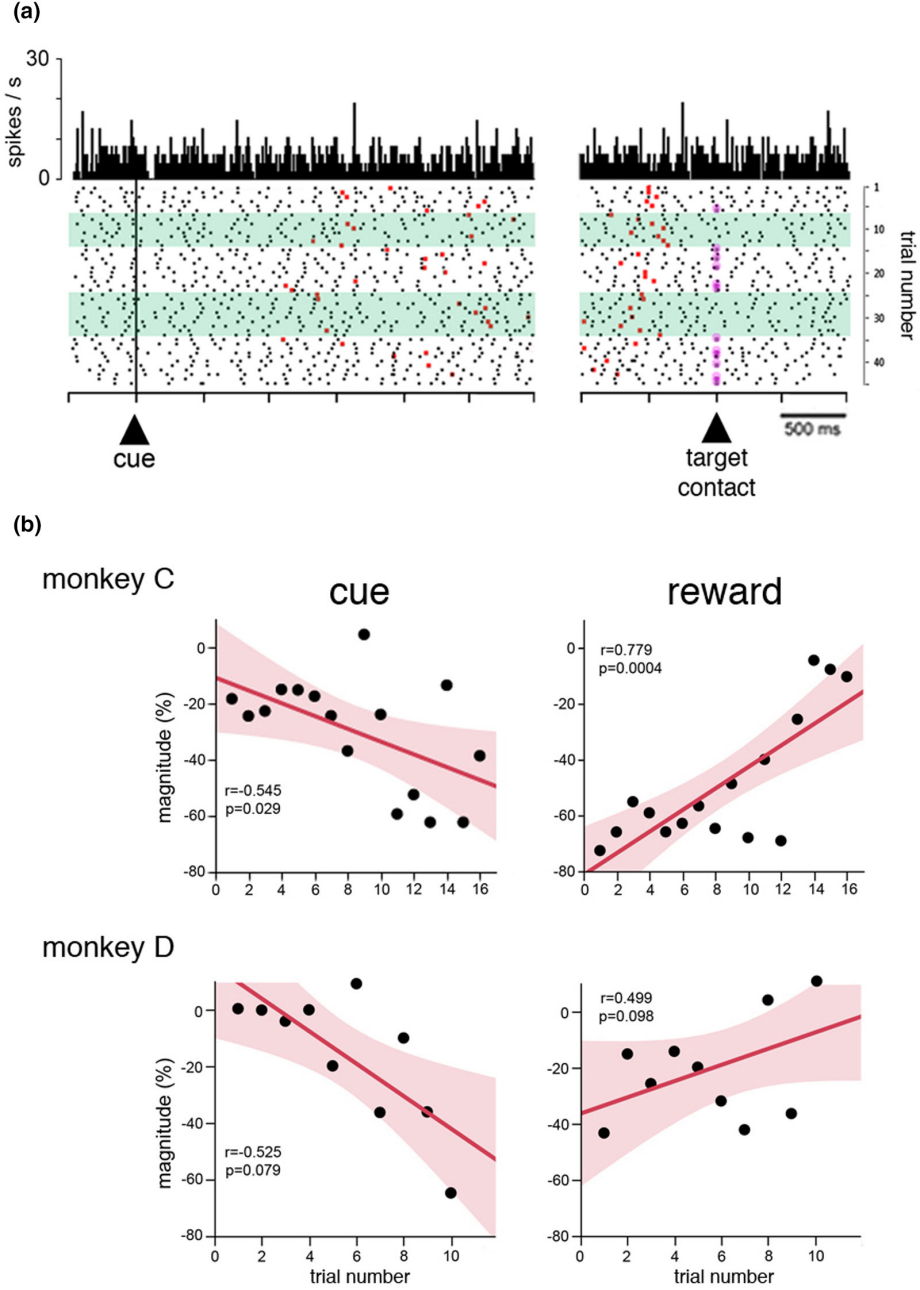
Influence of timing accuracy on TAN responsiveness. (a) Activity of a single TAN during performance of the timing task. The sequence of trials is shown chronologically from top to bottom in the raster display (45 trials total). Green shading shows sequences of correctly timed movements performed repeatedly for more than 5 trials (trials 7–14 and 25–34). Same conventions as in Figure 2b, except that purple dots aligned on target contact indicate unrewarded trials (underestimation errors). (b) Trial‐by‐trial variation of TAN responsiveness to the cue and reward according to the trial number during sequences of correctly timed movements. Scatter plots of the magnitude of TAN modulations (ordinate) versus the number of consecutive correct trials (abscissa). Same conventions as in Figure 5a, except that each point corresponds to the average across neurons in each trial of the sequence.

We next asked whether the sensitivity of the TANs to the timing cue and reward could be hidden by fluctuating performance in producing correctly timed movements from trial to trial. We then specifically examined TAN activity during periods of stable timing performance, namely when the monkeys performed sequences of at least five consecutive correct trials. For this analysis, we combined data from both short and long intervals in order to maintain the chronological order of the trials. The results revealed different degrees of stability in timing performance between the two animals, monkey C being able to make up to 16 correctly timed movements over consecutive trials, whereas monkey D did not exceed 10. Figure 6a shows an example of a TAN recorded during the course of a block containing two sequences of correctly timed movements. We focused our analysis on these sequences and we computed, trial‐by‐trial, the magnitude of TAN modulation. We then conducted linear regression analyses of the magnitudes of TAN responses to the cue and reward as a function of trial number in the sequence. A summary of this analysis for the two monkeys is shown in Figure 6b. In monkey C, we found that cue and reward responses were significantly correlated with the trial number, albeit in opposite manners, namely the correlation was negative for the cue response and positive for the reward response (Figure 6b, upper panels), indicating that TAN responses to the cue became larger across successive correctly timed movements as TAN responses to reward became weaker. The same trend was apparent in monkey D (Figure 6b, lower panels) but there were too few trials in sequence to detect statistically significant differences in activity. It therefore appears that the responsiveness of TANs seemed to scale with the trial number when monkeys maintained a steady level of correct performance, indicating that the neurons were sensitive to the degree of success in obtaining reward as a reflection of its predictability.

To summarize, we found that TAN responses to the cue and reward changed on a trial‐by‐trial basis when monkeys performed the task well, the cue response increasing and the reward response decreasing during periods of stable timing performance. This effect was obvious in monkey C and less apparent in monkey D, possibly because of the relatively small number of consecutive correct trials which dampened the effect of stable timing performance in this latter animal. It therefore appears that trial type history influenced the neuronal responsiveness, in relation to an evolving estimation of the rates of reward experienced over successive trials.

### 3.7 Influence of interval duration in the Pavlovian protocol

**FIGURE 7:**
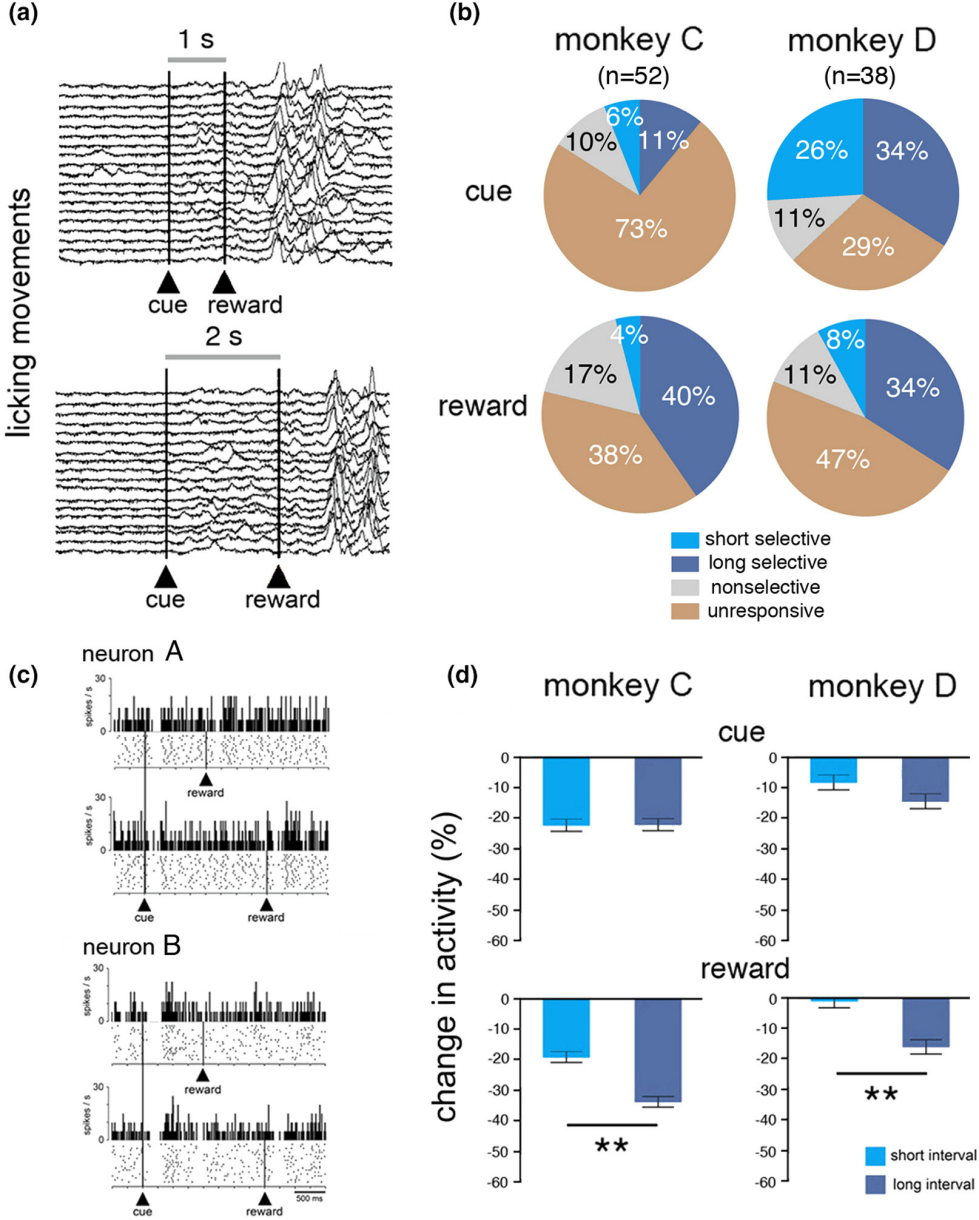
Effects of interval duration on TAN activity in the Pavlovian conditioning task. (a) Licking movements after the onset of the cue. Traces of lick records are aligned on the cue onset for each interval duration. Licking started immediately after the presentation of the cue, indicating that animals have learned the reward predictive value of the stimulus. (b) Percentages of different response types evoked by the cue and reward. (c) Two example neurons displaying responses to the cue and reward. For each neuron, the top panel shows short‐interval trials and the bottom panel long‐interval trials. Same conventions as in Figure 3a. (d) Comparison of magnitudes of changes in TAN activity after the cue and reward separately for short‐ and long‐interval trials. **p < 0.01 (Wilcoxon signed‐rank test).

In addition to recording the TANs in the TET, we tested a sample of them in a PCT in which the cue solely indicated the timing of reward, with no requirement for controlled temporal processing. We used two time intervals in the same range as those used in the TET and same relationships between cue location and interval duration, thus allowing us to further examine to what degree temporal information processing and reward prediction could influence TAN activity. In the PCT, both monkeys showed anticipatory licking movements during the interval between stimulus and reward, thus reflecting the reward‐predictive value of the cue (Figure 7a). Among 90 TANs tested in this condition, responses to the cue and reward occurred in 41 neurons (monkey C: 14/52, 27%; monkey D: 27/38, 71%) and 52 neurons (monkey C: 32/52, 62%; monkey D: 20/38, 53%), respectively. The fraction of neurons responding to the cue was higher in monkey D than in monkey C (*χ*2 = 17.23, *df* = 1, < 0.0001), whereas the fractions of neurons responding to reward were not different between animals (*χ*2 = 0.71, *df* = 1, *p* = 0.398). As shown in Figure 7b, there were no significant differences in the proportion of cue responses selective for one interval in monkey C (*χ*2 = 1.90, *df* = 1, *p* = 0.295) and monkey D (*χ*2 = 0.56, *df* = 1, *p* = 0.453). In contrast, the percentage of selective responses to reward was higher for the long interval than for the short one in both monkeys (monkey C: *χ*2 = 20.15, *df* = 1, *p* < 0.0001; monkey D: *χ*2 = 7.91, *df* = 1, *p* = 0.004), in accordance with the notion that the timing of reward was less well predicted in long‐interval trials, compared to short ones. Examples of neurons responding to the cue and reward in the PCT are shown in Figure 7c. The first neuron (top) showed a response to the cue and reward regardless of interval duration. The second neuron (bottom) showed a response to the cue that was invariant to the interval duration, whereas response to reward was stronger for the long interval.

We conducted the same analysis as that done in the TET to assess quantitatively the effects of interval duration on changes in TAN activity within time windows determined for each event and each monkey. As indicated in Figure 7d, the magnitude of TAN response to the cue did not show any significant difference between the two intervals (monkey C: *z* = −0.436, *p* = 0.662; monkey D: *z* = 1.758, *p* = 0.078), whereas a modulation of reward response depending on interval duration emerged in both monkeys, with stronger responses to the long interval compared to the short one (monkey C: *z* = 5.500, *p* < 0.0001; monkey D: *z* = 4.508, *p* < 0.0001).

It therefore appears that the time elapsed from the cue onset in the PCT influenced the responsiveness of TANs to the subsequent reward in both monkeys, with larger responses when the predicted timing of reward was assumed to decline as interval duration increased. This time‐dependent effect was in line with our prior studies showing that delayed reward timing in a Pavlovian procedure has an enhancing effect on the TAN response to reward (Apicella et al., 1997; Ravel et al., 2001). On the other hand, the magnitude of the TAN response to the cue predicting reward did not changed, confirming previous results based on reward probability manipulations (Apicella et al., 2009).

### 3.8 Sensitivity of TANs to the cue within distinct striatal regions

**FIGURE 8:**
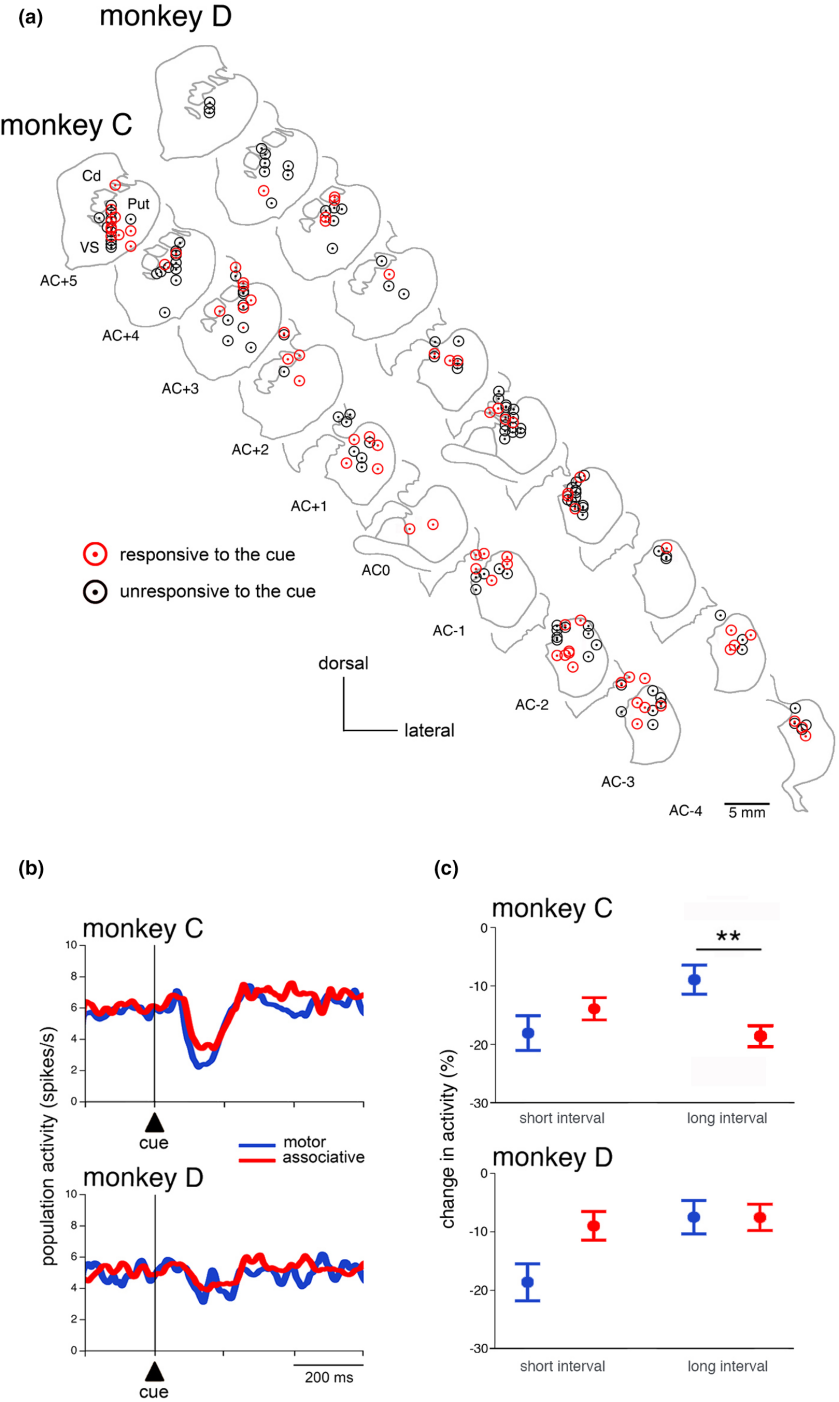
Sensitivity of TANs to the timing cue among striatal regions. (a) Recording sites of TANs responding to the cue in both monkeys. The location of all recorded TANs is plotted in the rostrocaudal direction on coronal sections from 5 mm anterior to 3–4 mm posterior to the anterior commissure (AC), with 1 mm intervals. Cd, caudate nucleus, Put, putamen. (b) Comparison of population activities of TANs aligned on cue onset, grouped for short and long intervals, between motor and associative striatum. Same conventions as in Figure 3d, except that curves are smoothed with a Gaussian filter (alpha = 0.04). (c) Differences in the magnitude of TAN responses to the cue between the motor and associative striatum according to interval duration (Wilcoxon signed‐rank test, *p* < 0.01).

In primates, the posterior putamen is related to motor functions while the dorsal part of the anterior striatum, including both the caudate nucleus and putamen, is involved in associative functions. In order to look for region‐specific signals in TANs, the location of the recorded neurons was identified histologically in both monkeys (Figure 8a). In all, we recorded from 80 and 120 TANs in the motor and associative striatum, respectively, and we compared TAN responses to the cue in the two regions. Responses to the cue were found in 43 neurons of the associative striatum (monkey C: 29/68, 43%; monkey D: 14/52, 27%) and 33 neurons of the motor striatum (monkey C: 22/46, 48%; monkey D: 11/34, 32%) and frequencies of cue responses did not vary significantly between the two regions (monkey C: *χ*2 = 0.298, *df* = 1, *p* = 0.585; monkey D: *χ*2 = 0.294, *df* = 1, *p* = 0.587). Data from the two monkeys were also analyzed at the level of the whole population of TANs sampled in each striatal region (Figure 8b). The responsiveness of TANs to the cue did not show evidence of preferential localization, fractions of responsive neurons being evenly distributed over the two dorsal striatal regions. Visual inspection of the population activity of TANs indicates that the average responses to the cue were comparable in the motor and associative striatum of each monkey, being much weaker in monkey D. Concerning a relationship to interval duration (Figure 8c), a higher responsiveness of TANs in the associative striatum of monkey C was observed only in response to the cue associated with the long interval (z = 3.461, *p* = 0.0005). This result reflects a difference in the sensitivity of TANs to interval duration depending on the striatal region.

## 4 DISCUSSION

Previous experimental studies and theoretical models assessing the role of the striatum in the processing of time have focused on output neurons and did not consider the contribution of local circuit neurons which are critical regulators of the striatal network activity (Buhusi & Meck, 2005; Merchant et al., 2013). Here we provide the first account of changes in the activity of TANs, presumed cholinergic interneurons of the striatum, while monkeys made self‐initiated movements based on time estimates. Behavioral data confirmed that the timing performance became more variable as interval duration increased, an effect known as the scalar property of timing (Gibbon, 1977), indicating that monkeys were able to adjust the timing of their movements according to temporal information contained in the cue. Over one‐third of the TANs we recorded displayed brief depressions in firing in response to the cue that defined the moment when monkeys could start the movement. By investigating the relationship between modulations of TAN activity and timing performance, we found that TANs could integrate temporal and reward information in their responses. These findings reveal a new aspect of the information encoding capacity of the TAN system which extends to the ability to react in a temporally controlled manner.

### 4.1 Role of TANs in controlling timed movements

To perform the timing task correctly, monkeys must retrieve temporal information contained in a visual cue, wait for a specified time interval, and then initiate a movement to obtain reward. There are three findings in the present study indicating that TANs may be involved in the processing of information required for the initiation of self‐timed movements.

First, most TANs responding to the cue displayed responses differentiating the duration of the ensuing interval (64 of 74 responsive neurons, 86%). The selectivity of TAN responses when the cue was associated with a short or long time interval suggests that these responses may signal the detection of a stimulus which has been associated with a given duration to determine when to generate movements. Second, TAN responses to the cue were different between animals, monkey C being more successful in correctly estimating the timing of movement, as compared to monkey D. In parallel, the responsiveness of TANs to the cue was higher in monkey C (45%) than in monkey D (27%), these differences being unrelated to the anatomical location of the recording sites as TANs were sampled in the same dorsal striatal regions in both subjects. Third, in monkey D, the TAN response to the cue was stronger when it was associated with the short interval, the differential responsiveness being coupled with a tendency to initiate movements close to the time threshold for movement onset. As it will be discussed later, this could reflect a particular strategy for motor timing in short‐interval trials. Overall, these findings can be taken as evidence that TAN responses to the cue were influenced by temporal processing. To explore this question further, we examined the relationship between TAN responses to the cue and the precise moment when animals started the movement. We found that in some instances TAN responses to the cue were scaled by the timing of movement, namely the magnitude of TAN responses was stronger with earlier initiation of responding in short‐interval trials (monkey C) or stronger with later initiation of responding in long‐interval trials (monkey D), providing support for the idea that the level of TAN modulation is coupled to the estimated time of movement onset.

### 4.2 What signals are encoded by TANs during timing behavior?

Based on the present findings, we hypothesize that the TAN response to the cue may act as a starting signal initiating the timing process required for making self‐initiated movements. Electrophysiological studies in animals performing timing tasks have established that striatal encoding of temporal information in the range of seconds relies on dynamic changes in activity of populations of projection neurons. Indeed, several recording studies in rodents and monkeys have documented the involvement of striatal output pathways in encoding elapsed time, at both single‐neuron and population levels in a variety of TETs (Bakhurin et al., 2016; Bakhurin et al., 2017; Chiba et al., 2015; Gouvêa et al., 2015; Jin et al., 2009; Matell et al., 2003; Mello et al., 2015; Wang et al., 2018; Zhou et al., 2020). The TAN signal at the beginning of the interval to be timed could be integrated within the striatal circuitry, interacting with sustained and/or sequential activation patterns of the projection neurons that convey information about how much time has elapsed from the cue onset. This role is reminiscent of that proposed for the phasic activation of dopaminergic neurons projecting to the striatum in a theoretical model of interval timing, called the striatal beat frequency model (Buhusi & Meck, 2005; Matell & Meck, 2000; Matell & Meck, 2004). According to this model, the dopaminergic signal could serve as a trigger for estimating the duration of a stimulus or delay. One can speculate, based on known interactions between dopaminergic and cholinergic transmissions within the striatum (Cachope et al., 2012; Cai & Ford, 2018; Threlfell et al., 2012; Threlfell & Cragg, 2011), that signals carried by dopaminergic neurons and cholinergic TANs interact with timekeeping processes, possibly by regulating both synaptic plasticity and excitability of striatal output neurons involved in the representation of time elapsed from cue onset.

From an anatomical viewpoint, we found that TANs responsive to the cue were distributed across the dorsal part of the anterior putamen, a region subserving cognitive‐related functions, and the posterior putamen, which is involved in motor control, indicating that the signal emitted by TANs may exert a widespread influence on output pathways within the striatum. Several fMRI studies in humans reported activations in dorsal regions of the striatum, including the caudate nucleus and putamen, in many timing tasks (Coull et al., 2008; Coull et al., 2011; Harrington et al., 2004, 2010; Rao et al., 2001). Interestingly, in monkey C, the TAN responsiveness to the long‐interval cue was stronger in the associative striatum than in the motor striatum. This may reflect more cognitive demands linked with the associative striatum because of attentional load in long‐interval trials. It is therefore possible that the processing of interval durations recruits anatomically segregated striatal circuits that are distinctly modulated by TANs. A difference in TAN response properties dependent on striatal regions has previously been reported, with TAN responses to a movement‐triggering stimulus being more prevalent in the putamen than in the caudate nucleus (Yamada et al., 2004).

### 4.3 Interaction between time processing and reward prediction

The monkeys' accuracy at estimating the elapsed time at different intervals was determinant for deciding when to initiate movements to obtain reward. It is therefore conceivable that TAN activity produces an expectation of the outcome in addition to time processing. As the task we used did not allow independent variation of temporal and reward parameters and given our previous work that the sensitivity of TANs to reward is reduced when the probability or timing of reward is predicted (Apicella et al., 1997; Apicella et al., 2009; Ravel et al., 2001), we have examined a potential influence of the expectation of receiving a reward through TAN responses to the reward itself.

In the TET, the outcome of self‐initiated movements could not be predicted with certainty as it depended on the monkey's ability to provide accurate time estimates for movement initiation. Consequently, the effect of reward prediction can vary according to individual timing performance. As already mentioned, monkey D was least successful in obtaining reward in the task. Then if reward was less certain in this animal, TANs should display larger responses to reward reflecting a lower level of reward predictability. However, it is worth noting that the modulation of activity of the whole population of TANs in response to reward was stronger in monkey C (−63%) than in monkey D (−15%), which is surprising considering that this latter animal performed less well than monkey C. Then, if rewards were less well predicted due to a lower level of timing performance, TANs should theoretically display stronger responses to reward. However, the observed effects in monkey D did not fit with this idea, suggesting little contribution of reward prediction in this animal, probably because of an overall decrease of the motivational level in the task (see below). In addition, timing performance was weaker in long‐interval trials than in short‐intervals trials in both monkeys, which would be associated with a higher sensitivity of TANs to reward in long‐intervals trials. In support of this, we found that the strength of TAN responses to reward was enhanced with interval duration in monkey C which may be interpreted as an effect of reward prediction. What is less clear is why such an effect did not emerge in monkey D, the overall responsiveness to reward being surprisingly low in this animal, regardless of interval duration. There was also an effect of movement timing on the magnitude of TAN responses to reward for both intervals in monkey C, whereas such an effect was only observed for the long interval in monkey D. One possible explanation for the unexpected finding of an attenuation of TAN responses to reward in monkey D may be related to an overall decreased engagement in the task that interferes with the responsiveness of TANs. During training, it was noticed that this animal was not willing to make effort in the TET when we attempted to lengthen the interval beyond the 2 s duration, which might be interpreted as a low level of motivation possibly contributing to diminished TAN responding to reward.

The integration of time processing and reward prediction in TAN response is further supported by the results of our complementary analysis focusing on successive correctly timed movements. When monkeys repeatedly succeeded in obtaining reward in the TET, we observed a graded modulation in the TAN response to the cue and reward, likely reflecting monkeys' assessment of trial‐by‐trial cue values as a reward‐predicting cue. This was paralleled by a weaker response to reward across those trials, consistent with the idea that monkeys had acquired confidence in their ability to perform the task well thus allowing to better predict reward delivery. Overall, these findings provide support to the idea that the responsiveness of TANs was influenced not only by temporal variables, but also by predictable rewards, suggesting that reward prediction could be incorporated in TAN signals during timing performance.

Finally, to further investigate the interaction between time processing and reward prediction, we tested a subset of the neurons in the PCT, a much simpler condition in which reward was automatically delivered after a specified length of time elapsed from the cue. In this condition, the influence of interval duration on TAN responses to reward was consistent between monkeys, being stronger with the long interval than with the short one, in line with the notion that reward prediction decreases with the duration being timed (Apicella et al., 2009; Ravel et al., 2001). It is noteworthy that this effect only emerged for the reward and not for the preceding cue, consistent with our previous findings in a Pavlovian protocol showing an enhancement of the TAN response to reward as the probability of reward decreased, whereas the response to the reward‐predicting stimulus remained largely unaffected by changes in the probability of reward (Apicella et al., 2009).

Altogether, those results support the notion that reward prediction should be considered as a possible confounding factor influencing the responsiveness of TANs during timing behavior. A link between time estimation and reward prediction has been demonstrated for midbrain dopaminergic neurons in mice performing a temporal discrimination task (Soares et al., 2016). Neuroimaging studies in humans attempting to dissociate reward and timing processes have reported activations in striatal and prefrontal cortical areas that may reflect the integration of time perception and reward prediction (Tomasi et al., 2015). Theoretical models incorporating time as a crucial aspect of reward processing also emphasize the importance of motivational variables on timing processes (Daw et al., 2006; Ray & Bossaerts, 2011; Galtress et al., 2012; Gershman et al., 2014; Kirkpatrick, 2014; Namboodiri et al., 2014; Apaydin et al., 2018; Petter et al., 2018). Additional work using behavioral procedures that will allow independent manipulation of temporal and reward parameters is needed to distinguish TAN signals specific to temporal processing from those related to reward prediction.

### 4.4 Role of the intrinsic striatal cholinergic system in the encoding of time

Prior pharmacological studies have emphasized the importance of brain cholinergic systems in timing functions (Meck, 1983, 1996). Disruption of timing behavior after systemic injections of drugs affecting cholinergic transmission has been interpreted as reflecting deficits in temporal memory and attention (Meck & Church, 1987) presumably as a consequence of reduced cholinergic input to the frontal cortex and hippocampus (Meck et al., 1987). The first evidence of a direct contribution of local cholinergic innervation of the striatum to time processing has come from a recent study in rats showing that a selective immunotoxin‐induced ablation of dorsal striatal cholinergic interneurons elicited an impaired acquisition of duration memory (Nishioka & Hata, 2024). Clinical studies have also shown deficits in the temporal control of motor behavior in patients with Tourette syndrome (Graziola et al., 2020; Schüller et al., 2021; Vicario et al., 2010) who have deficits in cholinergic signaling in the striatum (Kataoka et al., 2010).

Apart from the phasic changes in TAN activity, one cannot exclude that additional encoding mechanisms not studied here may impact striatal output pathways involved in the representation of time elapsed from cue onset. In particular, the level of synchronized firing among TANs could change through the time interval preceding movement initiation. Previous studies in macaques have shown that TANs can exhibit transient synchrony of firing during behavior (Kimura et al., 2003; Raz et al., 1996) and recent work in mice using recording from populations of striatal cholinergic interneurons has reported modulations of the cholinergic tone across large regions of dorsal striatum during spontaneous movements (Howe et al., 2019). One can speculate that a tonic, rather than phasic, change in cholinergic tone may also play a role in modulating striatal dynamics supporting the generation of self‐initiated movements based on time estimates.

### 4.5 Study limitations

It is worth mentioning that the present study was subject to limitations due to task design restrictions. In the TET, monkeys reported their time estimates at two laterally located targets and it remains possible that visuospatial factors may impact timing performance and associated TAN responses to the cue. It has been shown that TANs can be involved in the processing of spatial attributes of stimulus and/or movement (Ravel et al., 2006; Shimo & Hikosaka, 2001) and their responses to task events have also been proposed to drive attentional shifts required for stimulus detection (Ding et al., 2010; Matsumoto et al., 2001; Stalnaker et al., 2016). In our experiments, attempts to counterbalance target locations and time intervals were not successful and we cannot rule out the possibility that spatial attributes and/or differences in the monkey's allocation of attention might influence the strength of TAN modulations. Although the integration of visuospatial information in the modulation of TAN activity remains an open question, it must be emphasized, however, that the reported TAN responses to spatially localized cues were not systematically stronger in the TET, assumed to be the more attention‐demanding task, compared to the PCT. This suggests that the differential responsiveness is not in a simple way related to different levels of attention.

Another potential limitation concerns the durations chosen to represent short and long intervals in the present study. Our findings indicate that the monkey's timing strategy might change according to the interval duration and this could influence TAN activity in a different way. As mentioned before, monkey D adopted a strategy leading to start movement close to the time criterion in short‐interval trials, possibly reflecting a delayed reaction to the cue rather than a decision rooted in time estimation. In this animal, the delay before reaching the time criterion exactly matched the duration of cue presentation (i.e., a 0.5 second long stimulus followed by a 0.5‐s delay) which might have biased the monkey toward using the presentation of the cue as a visual template to wait for the required duration in a more accurate manner. According to this view, movements in short‐interval trials can be assumed to engage a reactive mode of motor timing control. It would be interesting to examine TAN properties when monkeys performed the timing task using a broader range of intervals, putting more constraints upon time estimation. Future research needs to disambiguate the effects of different timing strategies from those specific to temporal processing per se.

## 5 CONCLUSION

Our view of TAN function in time perception is that these neurons act like detectors of stimuli relevant for initiating a timing process. Additional research is needed to characterize cholinergic signaling within the striatum during the performance of tasks in which timing processes may be better isolated from other confounding processes, such as reward prediction and attention. Given the cooperative role of acetylcholine and dopamine in the regulation of corticostriatal synaptic plasticity, it is important to consider that cholinergic TANs may be central to the expression of timing behavior. It is still unclear how dopamine‐acetylcholine interactions in the striatum contribute to the encoding of time which ultimately influences behavior. In particular, dopaminergic neurons send information to widespread regions at both cortical and subcortical levels, whereas the influence of cholinergic TANs remains confined within the striatal circuitry, suggesting that their contribution may not be equivalent. A critical next step will be to understand the respective roles of these two modulatory systems in the striatal mechanisms of timing behavior.

## FUNDING INFORMATION

This work was supported by Centre National de la Recherche Scientifique and the Association France Parkinson. Funding for A. C. Martel was provided by a doctoral fellowship from the French government, Fondation pour la Recherche Médicale, and Fondation des Treilles.

## ETHICS STATEMENT

Experimental protocols were in accordance with the National Institutes of Health’s *Guide for the Care and Use of Laboratory Animals* and approved bythe Ethics Committee of the Institut de Neurosciences de la Timone (protocol#3057‐2015120809435586), in compliance with the French laws on animal experimentation.

## Data Availability

Data will be made available upon request.
